# Incidental pulmonary emboli in stage IV melanoma patients: Prevalence in CT staging examinations and improved detection with vessel reconstructions based on dual energy CT

**DOI:** 10.1371/journal.pone.0199458

**Published:** 2018-07-12

**Authors:** Monika Uhrig, David Simons, Heinz-Peter Schlemmer

**Affiliations:** German Cancer Research Center (DKFZ), Department of Radiology, Heidelberg, Germany; University of Queensland Diamantina Institute, AUSTRALIA

## Abstract

**Objectives:**

Malignancy is the strongest predictor for venous thromboembolism. Dual energy CT (DECT) can support assessment of pulmonary emboli (PE) by providing vessel reconstructions (DECT-VR) and highlighting thrombi. Purpose was to determine prevalence and risk factors of PE in staging examinations of stage IV-melanoma patients and to evaluate the potential of DECT-VR to improve PE diagnosis.

**Material and methods:**

This retrospective study was approved by IRB. Contrast-enhanced, conventional grey scale CT (cCT) and DECT-VR of 200 stage IV-melanoma patients were reviewed by three radiologists in consensus. Overall prevalence was determined. One-sided Wilcoxon-test was performed to compare the number of detected emboli between cCT and cCT with supplementary DECT-VR. Frequencies of risk factors were compared with χ^2^ test.

**Results:**

On cCT, 9 PE were detected (6 patients, correlating to 3% of the study population with 0.05 emboli per patient). With the supplementary DECT-VR, number of diagnosed emboli increased from 9 to 17 (p < 0.05) (in total 9 patients, correlating to 0.09 emboli per patient). Emboli on DECT-VR were mainly subsegmentally (7 of 8). There was no significant difference in the frequency of risk factors.

**Conclusions:**

The prevalence of pulmonary emboli in our cohort of 200 stage IV melanoma patients was 5%. DECT-VR improved significantly diagnosis of PE, especially when located subsegmentally.

## Introduction

Malignancy is by far the strongest predictor for recurrent venous thromboembolism [1]. Pulmonary emboli (PE) has been reported to be the second leading cause of death among ambulatory cancer patients [2]. According to previous studies, a majority of deaths PE results from failure of diagnosis rather than failure of treatment [3, 4]. In a retrospective study of chest CT of oncologic patients with indications other than PE, 4% had pulmonary emboli, of which only 25% had been detected and reported initially [5]. This indicates the importance of an accurate radiological assessment.

Standard of care for cancer staging is computed tomography (CT). Dual energy CT (DECT) extends the diagnostic potential of conventional CT by complementing the anatomical knowledge with material-specific information. By using different x-ray spectra, materials with different atomic numbers can be separated [6]. Besides many clinical applications like characterization of renal stones or iodine quantification for therapy monitoring, DECT improves vessel visualization and identification of pulmonary emboli based on iodine analysis [7–10].

Animal studies demonstrate that DECT can achieve higher diagnostic accuracy for detecting peripheral pulmonary emboli [11]. Few studies indicate that DECT could improve detection of pulmonary emboli in humans as well [8, 12], especially when using (nonlinear) blending algorithms [13–15]. To the best of our knowledge, there are no studies evaluating vessel reconstructions based on material separation including large patient cohorts and focusing on staging examinations of oncology patients in clinical routine.

Purpose was to determine prevalence and risk factors of PE in 200 staging examinations of stage IV melanoma patients and to evaluate the potential of DECT vessel reconstructions to improve PE diagnosis.

## Material and methods

This retrospective study was approved by the institutional review board (Ethics Committee Heidelberg) and written informed consent was obtained from all patients.

### Patient population

200 contrast enhanced dual energy scans from 200 separate patients with stage IV melanoma (referring to the American Joint Committee on Cancer (AJCC classification)) were evaluated (83 female, 117 male patients). Mean age was 59 years, with a range of 26–87. All patients were outpatients. In this study, patients with malignancies other than melanoma have been deliberately excluded in order to assess the value of DECT in a homogeneous patient population. Furthermore, monitoring of innovative treatment in patients with advanced stage of malignant melanoma is one our research foci, and a considerable part of the staging examinations in our department are related to those patients.

Data acquisition included CT examinations from a time period of 16 months (June 2014 –September 2015).

Inclusion criteria were

- patient with histologically proven malignant melanoma stage IV
- protocol parameter as described in the following paragraph (staging examination, no PE protocol).

### CT examination and image reconstruction

Spiral image acquisition was performed on a second-generation 2 x 64-slice dual source dual energy CT (Siemens Somatom Definition Flash, Siemens AG, Forchheim, Germany), using two different tubes voltages (100 kV and tin filtered 140 kV, reference tube currents 185/143 mAs for thorax respectively 200/155 mAs for abdomen). The scan was acquired with online dose modulation (CARE Dose 4D, Siemens AG, Germany) and a detector collimation of 32 x 0.6 mm.

Examination protocol included intravenous application of nonionic iodinated contrast agent (Imeron 300, Bracco, Konstanz, Germany) with a body weight adapted amount and flow rate (Table 1) via an automated injector. Triggering contrast agent injection was realized by attenuation measurements in a region of interest (ROI) placed in abdominal aorta on the level of the liver hilus.

**Table 1:** Applied amount of contrast agent (Imeron 300, Bracco, Konstanz, Germany).

Body weight (kg)	Volume contrast agent (ml)	Flow rate (ml/s)
< 55	85	3.1
55–65	115	3.5
65–90	130	4
> 90	145	4.5

The CT´s evaluated in this study were part of a routine staging examination including an arterial imaging phase, analyzed in this study, and a portal-venous phase (both dual energy CT as mentioned above). Arterial phase started 10 seconds after the cut off value of 120 HU was detected (Bolus-tracking technique) with a caudocranial scanning direction and a pitch value of 0.9. Scan field of view was from neck to upper abdomen.

With a weighting factor of 0.5 the two datasets from the two x-ray tubes were fused to virtual images corresponding to a 120 kV scan (in the following these images will be referred to as “conventional grey scale CT” (cCT)) and were reconstructed into axial 3 mm slices with increment 1 mm using a standard soft tissue reconstruction kernel (D20f smooth). For lung parenchyma additional axial 1 mm slices were reconstructed using a standard very sharp reconstruction kernel (B70f very sharp).

DECT raw data from both x-ray tubes was also transferred to a dedicated workstation (SyngoMMWP VE36A, Siemens AG, Berlin and München, Germany). The software application named “Lung vessels” is based on a material decomposition selective for intravascular thrombotic material. The basic idea is comparing the x-ray absorption of the low (100 keV) and high (140 keV) energy spectrum, which is described by the ratio r. The default manufacturer preset of r = 1.09 was used for this study. The post-processing results in color-coded vessel reconstructions, where vessels with iodine are displayed in blue, soft-tissue or vessels without iodine due to emboli in red (in the following these images will be referred to as DECT vessel reconstructions).

### Data analysis

One radiologist with 5 years of experience in oncological imaging read the conventional CT images (B30f smooth kernel, 3 mm slices, window center/width 98/554 HU) first, followed by reading DECT vessel reconstruction images. Consensus reading with two additional radiologists was performed (4 and 15 years experience in oncological imaging). To diminish recall bias, CT evaluation for this study has been performed at least 9 months after the initial CT scan.

Standard criteria (e.g. arterial filling defect on at least two consecutive images, with sharp interface and surrounded by contrast agent, forming angles with the vessel wall) were used for identifying pulmonary emboli on conventional CT [16]. On DECT images, a red highlighted vessel section was only classified as pulmonary embolus when being retrospectively identified on conventional CT images (either 3 mm slices in B30f kernel or 1 mm slices in B70f kernel; “goldstandard”). The location of the diagnosed embolus was recorded and classified as either central, lobar, segmental or subsegmental.

To investigate potential risk factors for lung emboli, clinical notes were reviewed to report about medical cancer treatment (including cytotoxic chemotherapy, targeted therapy, immunotherapy), radiation therapy (ongoing or within 4 weeks before the CT examination), surgery within 4 weeks before the CT examination and ongoing anticoagulation. Laboratory results (measured within 4 weeks before the CT examination) were reviewed to determine platelet count and international normalized ratio (INR).

One-sided Wilcoxon-test was performed to compare the number of emboli detected on conventional grey scale CT to the number of emboli detected on grey scale CT with supplementary DECT vessel reconstructions, using MATLAB-software (MATLAB 2015a, The MathWorks, Inc., MA, USA). The frequencies of pulmonary embolus risk factors and abnormal laboratory test results in the patients with and without emboli were compared by using χ^2^ test. The statistical significance level was set to 0.05 for all analyses. For categorical data, distribution is given as percentage per category.

## Results

### Prevalence of lung emboli and increased detection by DECT vessel reconstruction

On conventional grey scale CT, 9 pulmonary emboli were detected (in 6 patients, correlating to 3% of the study population with 0.05 emboli per patient). With the supplementary DECT vessel reconstruction, number of diagnosed emboli increased from 9 to 17 (p < 0.05) (in total 9 patients, correlating to 0.09 emboli per patient).

**Fig 1:**
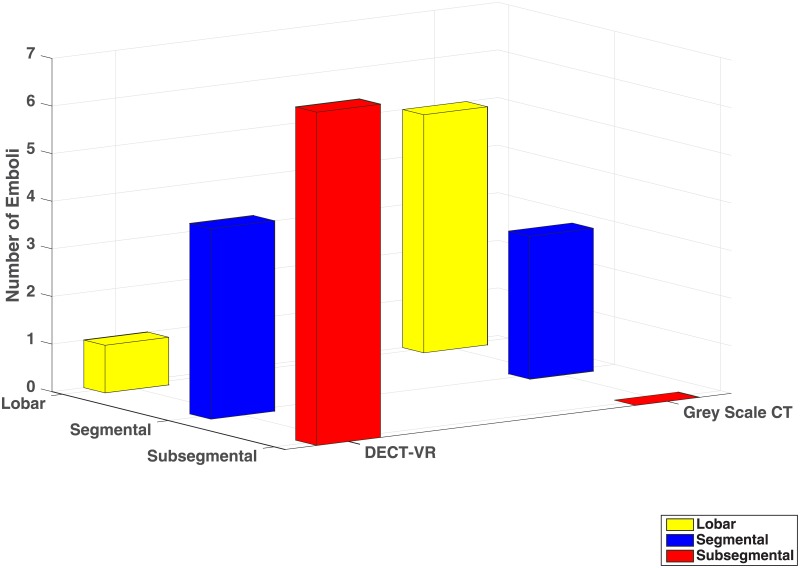
Number of Emboli (z-axis) detected on Dual Energy CT-vessel reconstructions (DECT-VR, bars on the left) and on conventional grey scale CT (bars on the right), subdivided into their anatomical location (yellow: lobar, blue: segmental, red: subsegmental). Emboli detected on DECT-VR were mainly subsegmentally located.

**Fig 2:**
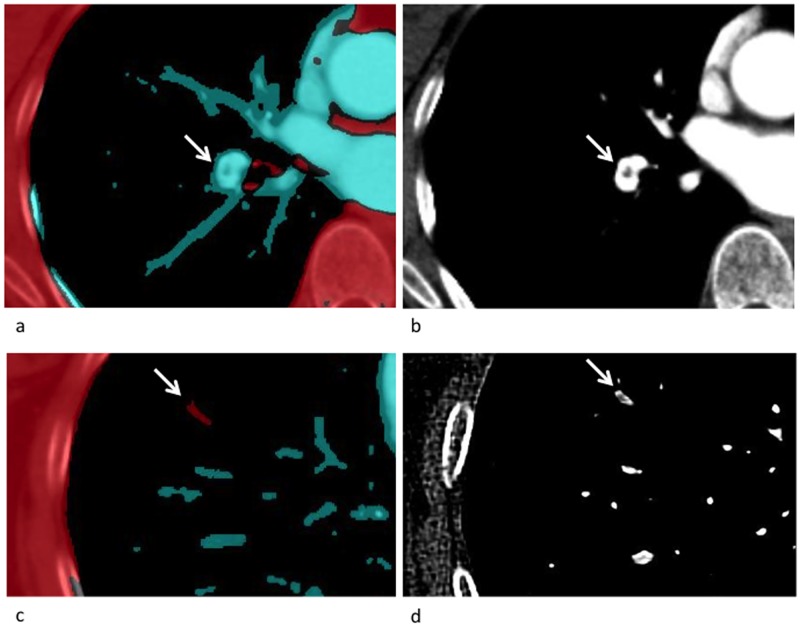
Grey scale CT and DECT vessel reconstruction from a female patient (75y) diagnosed with stage IV melanoma. A proximal embolus in right lower lobe was not highlighted by the DECT vessel reconstruction software (a) but reliably diagnosed on grey scale CT (b). A subsegmental embolus in right lower lobe was primarily detected in DECT vessel reconstruction (c), and could retrospectively be delineated in thin-slice (1 mm) reconstructions of the corresponding grey scale CT (d).

The emboli detected by the DECT vessel reconstruction were mainly subsegmentally located (7 of 8), while the emboli detected by the conventional CT could be found in more proximal arteries (segmental, lobar or central) (Fig 1, Table 2). 5 of 9 emboli diagnosed on grey scale CT were not highlighted in the DECT vessel reconstruction. They were exclusively located in lobar or central arteries (Fig 2, Table 2).

**Table 2:** Distribution and clinical features of incidental pulmonary emboli in stage IV melanoma patients.

Pat. Nr.	PE location	Reported on Greyscale	Reported on DECT	Treatment*	Platelet count*	INR*	Known history for thrombi or PE	Surgical History*
**1**	Segmental LLL	Yes	Yes	None	272	NA	Yes	No
	Segmental RUL	Yes	Yes					
	Lobar RLL	Yes	No					
	Segmental RML	No	Yes					
**2**	Subsegmental RLL	No	Yes	PD-1 Inhibitor	288	1.02	No	No
**3**	Subsegmental RLL	No	Yes	None	NA	NA	No	No
**4**	Subsegmental RLL	No	Yes	PD-1 Inhibitor	146	NA	No	No
**5**	Subsegmental RLL	No	Yes	BRAF and MEK Inhibitor	196	0.94	No	No
	Subsegmental RLL	No	Yes					
	Lobar RLL	Yes	No					
**6**	Right Trunk	Yes	No	Dacarbazin	733	0.99	No	No
	Lobar LLL	Yes	No					
**7**	Lobar RLL	Yes	Yes	None	247	0.94	No	Yes
**8**	Segmental RLL	Yes	Yes	PD-1 Inhibitor	NA	NA	No	No
	Subsegmental RLL	No	Yes					
	Subsegmental RLL	No	Yes					
**9**	Lobar LLL	Yes	No	Anti-CD20 Antibody	302	NA	No	No

* ongoing or within 4 weeks of CT examination; platelet count given in /nl with a normal range of 150-440/nl; normal range of INR 0.9–1.3; RUL = right upper lobe; RML = right middle lobe; RLL = right lower lobe; LUL = left upper lobe; LLL = left lower lobe; NA = information not available

**Fig 3:**
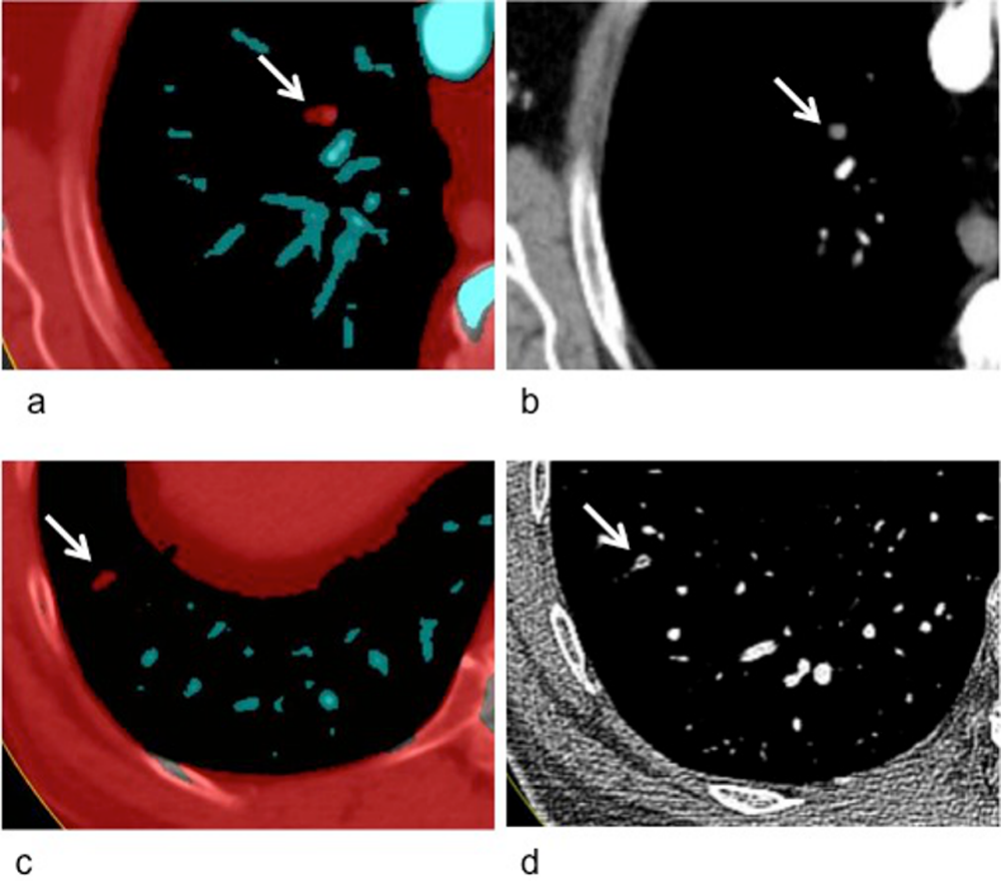
Pulmonary artery embolus in right upper lobe of a male patient (75y), clearly highlighted in dual energy CT (DECT) vessel reconstruction (a), but not detected on grey scale CT (b). Further pulmonary, subsegmental embolus in the right lower lobe of a male patient (77y), highlighted in red in the DECT vessel reconstruction (c). Retrospectively, a filling defect can be seen in thin-slice (1 mm) reconstructions of the corresponding grey scale CT (d).

Fig 3 demonstrates examples of emboli detected on DECT vessel reconstructions but not diagnosed on conventional grey scale CT.

**Fig 4:**
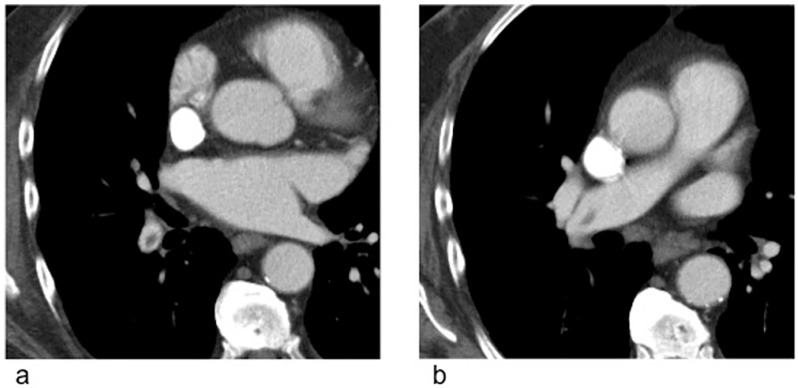
Follow up examination of patient Nr. 4 (see Table 2) 12 months later. While the CT evaluated in this study showed only a small subsegmental peripheral embolus (Fig 3c), the follow up CT demonstrated multiple lobar and central pulmonary emboli.

In summary, 3 patients were diagnosed with PE exclusively on DECT vessel reconstructions (patients Nr. 2,3 and 4 in Table 2). Clinical data about follow up of these patients was partially available:

Patient Nr. 2 was diagnosed 3 months later with an asymptomatic segmental PE and 5 months later with another asymptomatic segmental PE (both CT examinations were performed in external hospitals). The referring onocologists decided to start anticoagulation.
Patient Nr. 3 had no record for a PE (clinical information available for 12 months after the CT included in this study).
Patient Nr. 4 was diagnosed 8 months later with asymptomatic but multiple central and lobar emboli on the right (Fig 4) and was hospitalized for anticoagulation.

### Clinical history and risk factors

Table 2 lists all patients diagnosed with pulmonary emboli, including oncological treatments within 4 weeks before the CT examination, platelet count and INR (as far as available) as well as potential history of surgery and thrombi or emboli.

There was no significant difference in the frequency of the evaluated risk factors between the patients who did and the patients who did not have pulmonary emboli. Frequencies of medical cancer treatment, radiation therapy, history of surgery, thrombi or anticoagulation as well as platelet count and INR are given in Tables 3 and 4.

**Table 3:** Potential risk factors for pulmonary emboli (PE).

	All patients* (n = 200)	No. of Patients with PE* (n = 9)	No. of Patients without PE* (n = 191)	p**
Medical cancer treatment***	102 (51)	6 (67)	96 (50)	0.34
Radiation therapy****	10 (5)	1 (11)	9 (5)	0.4
**Surgery within 4 weeks of CT**	9 (5)	1 (11)	8 (4)	0.33
**Ongoing anticoagulation**	4 (2)	0 (0)	4 (2)	0.66

* numbers in parentheses are percentages (rounded to integers) relative to the total number of patients given in first line.

** p values based on χ^2^ test comparing frequencies of the given risk factor between patients with and without PE.

*** including cytotoxic chemotherapy, targeted therapy, immunotherapy, ongoing or within 4 weeks of CT

**** ongoing or within 4 weeks of CT

**Table 4:** Platelet counts and international normalized ratio (INR) (measured within 4 weeks before the CT examination).

	Total No. of patients* (n = 200)				No. of Patients with PE* (n = 9)				No. of Patients without PE* (n = 191)			
	Below Normal	Normal	Above Normal	NA	Below Normal	Normal	Above Normal	NA	Below Normal	Normal	Above Normal	NA
Platelet count**	8 (4)	114 (57)	11 (6)	67 (34)	1 (11)	5 (56)	1 (11)	2 (22)	7 (4)	109 (57)	10 (5)	65 (34)
INR***	1 (0)	58 (29)	3 (2)	138 (69)	0 (0)	4 (44)	0 (0)	5 (56)	1 (0)	54 (28)	3 (2)	133 (70)

* Number in parentheses are percentages (rounded to integers) relative to the total number of patients in the corresponding subgroup as given in the first line.

** Normal range for platelet count was 150–440 /nl

*** Normal range for international normalized ratio was 0.9–1.3

Note: χ^2^ test for comparing frequencies of abnormal test results between patients with and without PE resulted in no significant differences

NA = laboratory test result not available

PE = Pulmonary Embolus

## Discussion

Oncology patients have a considerably elevated risk for recurrent venous thromboembolism [17]. In clinical routine, small PE can easily be missed, particularly concerning peripheral located emboli [5, 18]. Although there is a controversial discussion about the clinical relevance of subsegmental PE [19, 20], recent studies indicate, that these patients mimic patients with more proximally located PE. Their risk profile and mortality risk differ significantly from patients without PE [17]. This underlines the relevance of accurate assessment of even small, peripheral PE. However, it has to be noticed that, in contrast to our study, the mentioned previous study evaluated patients with suspected PE, only a small percentage of them with a known history of malignancy [17]. In contrast, our study cohort consists exclusively of patients with advanced stage of malignant melanoma with a different balance of risks and benefits of anticoagulation. The clinical relevance of subsegmental emboli in asymptomatic cancer patients cannot be derived from our data and should be further investigated in a prospective study. However, 2 of 3 patients diagnosed with subsegmental PE in our study were diagnosed with more proximal located PE 3 to 12 months after the CT evaluated in this study.

### Detection of lung emboli with DECT

In our retrospective study with 200 stage IV melanoma patients, the number of diagnosed PE increased significantly by supplementary reading DECT vessel reconstructions. Most additionally diagnosed emboli were located subsegmentally. In line with our results, animal studies and studies with smaller subsets of patients with suspected pulmonary emboli have reported an increased sensitivity of DECT vessel reconstructions for diagnosis of PE, in particular for peripheral emboli [8, 11, 12, 21, 22]. To our knowledge, no studies have been published evaluating DECT vessel reconstructions in a large patient cohort and focusing on routine staging examinations of outpatients.

Five of nine proximal emboli detected on grey scale CT were not highlighted by the DECT vessel reconstruction. Insufficient visualization of proximal emboli has been described before and can be explained by the software algorithm being optimized to highlight thrombi in small peripheral vessels [8]. In a clinical setting, DECT vessel reconstructions should therefore rather supplement than replace conventional grey scale CT.

### Prevalence and risk factors

In this study, the prevalence of patients with PE was 5% (based on conventional CT with additional DECT vessel reconstructions). The prevalence of incidental PE in unselected cancer patients is reported slightly lower between 2 to 4% [23, 24], which is comparable to our result of 3%, when exclusively reading the conventional CT, without additional DECT vessel reconstructions. Gladish et al determined a comparable prevalence of 4% in a retrospective study of 403 oncology patients with several malignancies [5], while prevalence in the subgroup of melanoma patients (without specifying disease stage) was 10% (4 of 41 patients). The difference to the prevalence of 5% determined in our study could be related to the different size of patient population (41 vs. 200 melanoma patients).

Regarding the potential risk factors there were no significant differences, which can be confirmed by our results. It has to be noticed that the clinical information in our study was not complete for all patients, at least one of the evaluated risk factors was not available for 5 of 9 patients. Furthermore, the patients in this study cohort had no symptoms of acute pulmonary embolism. Those patients would have likely undergone immediate dedicated imaging. Overall, these issues could certainly affect the correlation between PE and potential risk factors evaluated in this study.

Going in line with our study, Gladish et al described that PE initially not reported in clinical CT were typically located in smaller arteries.

### Confounding imaging features and study limitations

There are several possible limitations to our study. Given the retrospective setting of the study, there was no histological confirmation of the emboli. This would be of interest because highlighted vessels can represent an embolus even if there is no correlation in conventional CT, which was shown in animal studies with histopathological confirmation [11]. The reference standard in our clinical study was consensus reading of three radiologists. An independent reading process by several radiologists would strengthen results [25].

Based on inheritant algorithm characteristics, not exclusively emboli in pulmonary arteries are highlighted in red. The software cannot differentiate between arteries and veins, furthermore soft tissues like pulmonary metastases, bronchus wall thickenings and consolidations are highlighted as well. In this context, the number of false positive findings was not determined in this study.

A technical limitation results from the field of view (FOV) of the two x-ray tubes of the dual source CT, which is not completely overlapping. In the corresponding scan area, spectral information being indispensable for DECT vessel reconstruction is missing, and peripheral lung parenchyma for larger patients could not be evaluated.

The influence of slice thickness, window setting and iodine content of vessels was not investigated in our study. In previous studies, thin-collimation and using wide window settings for grey scale CT resulted in better diagnostic rates [26, 27]. Furthermore, the influence of vessel iodine content was not investigated, which can influence diagnostic rates as well [28]. A low correlation between the correct PE diagnosis in DECT vessel reconstructions and the vascular attenuation has been described [8]. Finally this study did not include the monoenergetic reconstruction techniques provided by some DECT applications. Recent studies indicate that monoenergetic low-kV dual energy datasets as well as nonlinear blending algorithms significantly increase vessel attenuation [13, 29].

## Conclusion

In conclusion, the prevalence of pulmonary emboli in a set of 200 staging examinations of stage IV melanoma patients was 5%. DECT vessel reconstructions improved significantly diagnosis of incidental pulmonary emboli, especially when subsegmentally located. This could be of clinical importance considering that patients with clinically suspected PE diagnosed with subsegmental emboli have a significantly higher mortality compared to patients without PE, although this has not yet be shown for asymptomatic cancer patients.

## Abbreviations

cCT: conventional grey scale CT
DECT: dual energy CT
DECT-VR: dual energy CT vessel reconstructions
PE: pulmonary emboli
